# Molecular Paleoclimate Reconstructions over the Last 9 ka from a Peat Sequence in South China

**DOI:** 10.1371/journal.pone.0160934

**Published:** 2016-08-09

**Authors:** Xinxin Wang, Xianyu Huang, Dirk Sachse, Weihua Ding, Jiantao Xue

**Affiliations:** 1 Laboratory of Critical Zone Evolution, School of Earth Sciences, China University of Geosciences, Wuhan, China; 2 State Key Laboratory of Biogeology and Environmental Geology, China University of Geosciences, Wuhan, China; 3 Institute of Earth and Environmental Science, University of Potsdam, Potsdam, Germany; 4 Organic Surface Geochemistry Lab, Section 5.1 Geomorphology, GFZ German Research Centre for Geosciences, Potsdam, Germany; Institute of Tibetan Plateau Research, CHINA

## Abstract

To achieve a better understanding of Holocene climate change in the monsoon regions of China, we investigated the molecular distributions and carbon and hydrogen isotope compositions (δ^13^C and δD values) of long-chain *n*-alkanes in a peat core from the Shiwangutian (SWGT) peatland, south China over the last 9 ka. By comparisons with other climate records, we found that the δ^13^C values of the long-chain *n*-alkanes can be a proxy for humidity, while the δD values of the long-chain *n*-alkanes primarily recorded the moisture source δD signal during 9–1.8 ka BP and responded to the dry climate during 1.8–0.3 ka BP. Together with the average chain length (ACL) and the carbon preference index (CPI) data, the climate evolution over last 9 ka in the SWGT peatland can be divided into three stages. During the first stage (9–5 ka BP), the δ^13^C values were depleted and CPI and *P*_aq_ values were low, while ACL values were high. They reveal a period of warm and wet climate, which is regarded as the Holocene optimum. The second stage (5–1.8 ka BP) witnessed a shift to relatively cool and dry climate, as indicated by the more positive δ^13^C values and lower ACL values. During the third stage (1.8–0.3 ka BP), the δ^13^C, δD, CPI and *P*_aq_ values showed marked increase and ACL values varied greatly, implying an abrupt change to cold and dry conditions. This climate pattern corresponds to the broad decline in Asian monsoon intensity through the latter part of the Holocene. Our results do not support a later Holocene optimum in south China as suggested by previous studies.

## Introduction

The Asian monsoon is one of the key components of the global climate system. Monsoon climates, and particularly the summer monsoon-derived rainfall, are crucial to terrestrial ecosystems and human societies in the East Asia [1]. A clear understanding of past changes in monsoonal climate and their drivers, especially during the Holocene, which is closely linked to human evolution and development, is essential to understand how the terrestrial ecosystems respond to the present climate changes and to predict their future adaptations.

Numerous attempts have been made to explore the evolution of Holocene climate in monsoonal China, however, they display different scenarios. Some studies argued that the Holocene optimum was asynchronous among monsoon regions in China [1,2]. In contrast, some other researches did not support a time-transgressive Holocene optimum in China. For an example, Shi et al. (1992) [3] concluded that the Holocene optimum in China occurred from 8.5 to 3 ka BP. Zhou et al. (2007) [4] reckoned that that the Holocene optimum appeared almost synchronous around 10–5 ka BP across the monsoon regions of China. Similarly, Zhang et al. (2011) [5] suggested that the Holocene climate changes were broadly synchronous across the monsoon regions with Holocene optimum occurring during 10.5–6.5 ka BP. Therefore, more records generated from different sedimentary settings and based on various proxies are needed. Such records, on one hand, can help us draw an overall picture of the climate changes in one certain area; on the other hand, can enable us to make spatial comparisons of the Holocene climate in monsoonal China based on similar archives and proxies, eliminating uncertain factors brought by different archives and proxies.

Over the last two decades, biomarkers have been widely applied in the reconstructions of the paleoenvironmental and paleoclimatic conditions. In particular, *n*-alkanes have attracted fervent attention, due to their ubiquity, relative ease of extraction and purification from sediments and stronger resistance to microbial degradation [6]. They are increasingly employed as powerful proxies in paleoenvironmental research, especially through the application of compound-specific carbon and hydrogen ratios (e.g. [7–9]). A combination of *n*-alkane δ^13^C and δD analyses has been proven to be able to yield particularly useful information on vegetation and hydroclimate (e.g. [10–12]).

Peat deposits are ideal archives for molecular paleoclimate reconstructions in late Quaternary, due to their good capacity to preserve organic matter, successive sedimentation and sensitivity to hydrological oscillations [13,14]. Such deposits consist mainly of organic material, so that molecular paleoclimate proxies, in particular those derived from lipid biomarkers and their compound-specific isotope ratios, are suitable to extract paleoclimatic information [13,14]. Chinese peat deposits from the Holocene have received more and more attention in molecular paleoclimate studies over the last decade. Researchers have investigated peat deposits in northeast China [15–18], southwest China [19–22], central China [23–26], and south China [27, 28] with lipid biomarker approaches. However, studies of *n*-alkane δ^13^C and δD values in peat from south China are relatively few.

Here, we reported a molecular paleoclimate record from the Shiwangutian (SWGT) peatland, a previously unstudied peatland in south China. In this study, we investigated the distributions and δ^13^C and δD values of long-chain *n*-alkanes from the SWGT peat core over the last 9 ka. The *n*-alkane records were compared with pollen record from nearby Daping Swamp (~30 km away) [29], *n*-alkane δD record from Hongyuan peat sequence, southwest China (~1000 km away) [19], stalagmite δ^18^O (δ^18^O_carb_) record from Dongge Cave situated about 300 km to the southwest [30] and fatty alcohol record from the Dajiuhu peatland, central China (~600 km away) [24] to decipher the paleoclimatic significance of long-chain *n*-alkane δ^13^C and δD values and further to assess the paleoclimate changes for the last 9 ka in the SWGT peatland, south China, which can provide new evidence on the climate evolution during the Holocene.

## Materials and Methods

### Studying site

The SWGT peatland is located in the Chengbu Miao Autonomous County of Hunan Province, bordering the Ziyuan County of Guangxi Zhuang Autonomous Region. This region is affected by both the East Asian monsoon and Indian monsoon and is featured by a subtropical cool and humid mountain climate. The annual average temperature of this area is 13°C and the annual average precipitation is about 1800 mm [31]. The modern vegetation in this site is dominated by *Sphagnum* spp. and sedges, together with some bamboo species [31].

### Ethics Statement

All necessary permits were obtained for the described field work and were granted by the Administration of Shiwangutian Wetland Nature Reserve of Chengbu County. The field work did not involve endangered or protected species.

### Sampling

**Fig 1:**
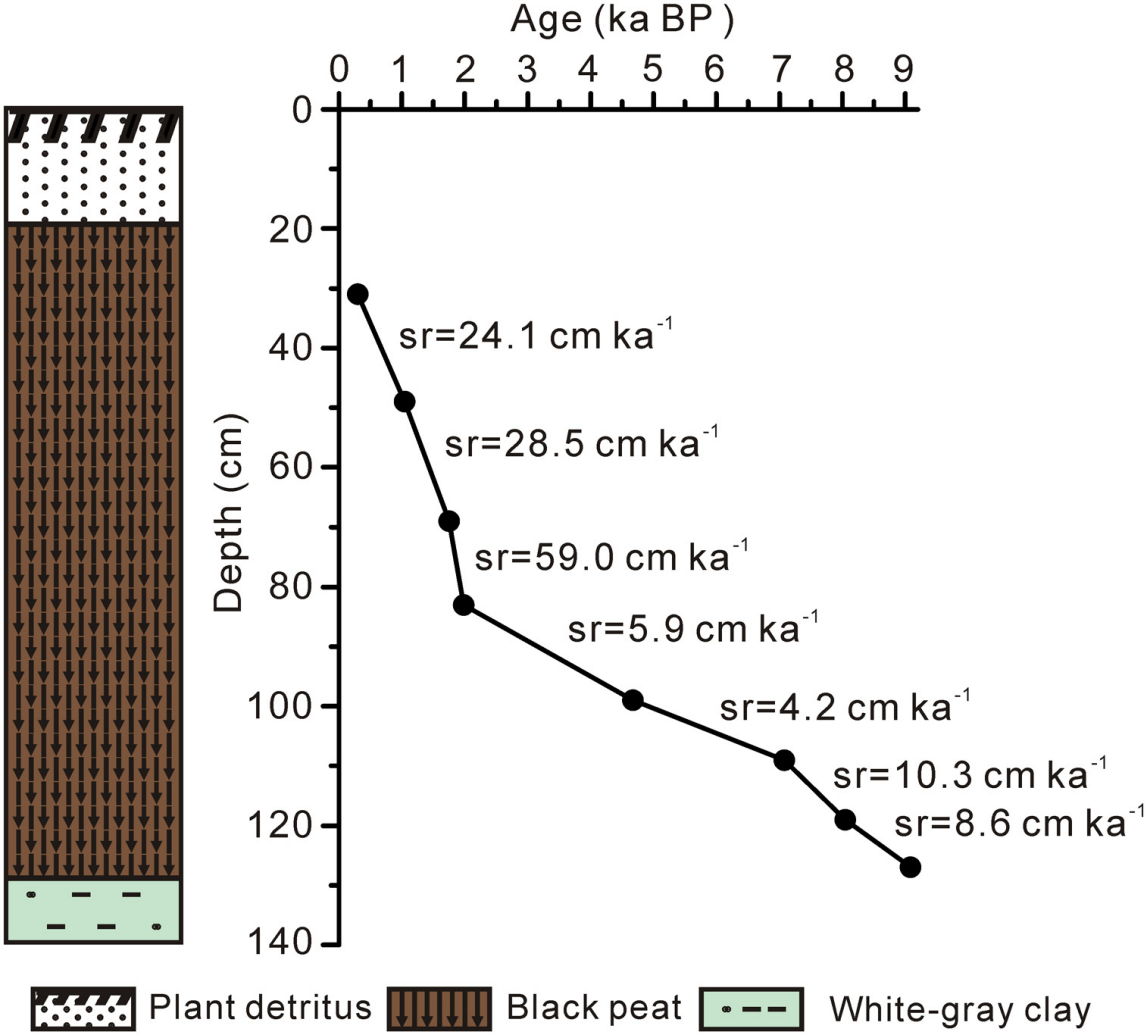
Age-depth plots and lithostratigraphic description for the SWGT peat core. Age control points (black dots) were obtained using the radiocarbon method. Average linear sedimentation rates (sr) are also shown.

A 1.4 m long peat core (26°05.20′ N, 110°21.98′ E; 1688 m above sea level) was retrieved in July 2012. Lithology of the SWGT peat core consists of an upper plant debris layer (0–20 cm), a black peat layer (20–128 cm) and a basal white-gray clay layer (128–140 cm) (Fig 1). Peat samples were collected at 2 cm interval. Samples for lipid analyses were from the upper 30–128 cm in this study.

Chronology of the SWGT peat core is based on AMS ^14^C dating of bulk peat. Calibrated age ranges were obtained using the Calib 7.0 software [32]. The age model is based on linear interpolation of paired calibrated ages.

### Lipid extraction and analyses

The freeze-dried peat samples were ground and sieved through a 60-mesh sieve (mesh aperture 0.3 mm). About 1.0 g peat was ultrasonically extracted 6×10 min with dichloromethane/methanol (DCM/MeOH) (9:1, v/v) after adding a mixture of internal standards (androstane, androstanol and cholanic acid). After solvent removal under reduced pressure, the extracts were fractionated into aliphatic, aromatic and polar fractions using silica gel column chromatography with hexane, hexane/DCM (1:1, v/v), DCM/MeOH (1:1, v/v) as the respective elution solvents.

Gas chromatography–mass spectrometry (GC-MS) analysis of the aliphatic fractions was performed on a Hewlett Packard 6890 gas chromatograph interfaced with a Hewlett Packard 5973 mass selective detector in the State Key Laboratory of Biogeology and Environmental Geology, China University of Geosciences. The chromatograph was equipped with a DB-5 column (30 m×0.25 mm, film thickness 0.25 μm). The oven temperature was programmed from 70°C (held 1 min) and then ramped to 210°C at 10°C min^-1^ and further increased to 300°C at 3°C min^-1^ (held 25 min). The identification of *n*-alkanes was achieved by a combination of GC retention times relative to standards and a mass spectrum library (NIST 08).

The quantification of the aliphatic fractions was performed on a Shimadzu GC 2010 equipped with a flame ionization detector (FID) and a DB-5 column (30 m×0.25 mm, film thickness 0.25 μm) in the State Key Laboratory of Biogeology and Environmental Geology, China University of Geosciences. The temperature program was identical to that of GC-MS. The absolute abundances were obtained by comparisons of peak areas with those of internal standards and adjusted with the relevant response factors.

### Compound-specific hydrogen and carbon isotope analyses

The δ^13^C values of long-chain *n*-alkanes were measured on a Finnigan Trace GC coupled to a Finnigan Delta XP isotope ratio mass spectrometer (GC-IRMS) in the State Key Laboratory of Biogeology and Environmental Geology, China University of Geosciences. Samples were injected in splitless mode (1 μl), with the injector temperature at 300°C. Separation was achieved by a DB-5MS column (60 m×0.25 mm i.d., film thickness 0.25 μm). The GC oven temperature was initiated at 50°C (held 1 min) and then ramped to 210°C at 10°C/min (held 2 min) and further raised to 300°C at 4°C/min (held 2 min) and finally increased to 310°C at 10°C/min (held 25.5 min). Helium was used as the carrier gas (1.0 ml/min). Instrument performance was checked every four runs using an *n*-alkane mixture with known δ^13^C values obtained from Arndt Schimmelmann (Indiana University, U.S.A.). Reproducibility for specific compounds was better than 0.5‰ (standard deviation), based on at least duplicate analyses. Results were reported in the δ notation (‰) relative to the Vienna Peedee Belemnite (VPDB) standard.

δD analyses of long-chain *n*-alkanes were achieved using Trace GC coupled with a Delta V advantage isotope ratio mass spectrometer in the State Key Laboratory of Biogeology and Environmental Geology, China University of Geosciences. Samples were injected in splitless mode (1 μl), with the injector temperature at 300°C. The GC oven temperature was programmed from 50°C (held 1 min) to 210°C at 10°C/min (held 2 min) and then to 300°C at 6°C/min (held 2 min) and finally to 310°C at 10°C/min (held 25 min). The high temperature conversion (HTC) system was operated at 1400°C and the HTC tube was conditioned with methane. H^3+^ factor was monitored daily, through the measurement period, its values varied around 3.7 and 4.2. To check the system stability, an *n*-alkane mixture (*n*-C_23_, *n*-C_25_, *n*-C_27_, *n*-C_29_ and *n*-C_31_ alkane) and the Indiana A4 mixture with known δD values were run between every two samples. Squalane (δD = 167‰) was used as the internal standard. Reproducibility for specific compounds was better than 5‰, based on at least duplicate analyses. All δD values are reported in the δ notation (‰) relative to the Vienna Standard Mean Ocean Water (VSMOW) standard.

### Calculations and statistics

The new time series of δ^18^O_carb_ record from Dongge Cave were obtained by Matlab R2011b software (The MathWorks Inc., U.S.A.) through cubic spline interpolation method, sharing a common time vector with *n*-C_29_ δD (δD_C29_) record from the SWGT peatland. The correlation analysis between δD_C29_ record from the SWGT peatland and new time series of δ^18^O_carb_ record from Dongge Cave was performed with SPSS 16.0 software (International Business Machines Co., U.S.A.).

## Results

### Chronology

Radiocarbon dating results are listed in Table 1 and also shown in Fig 1. Based on the age-model, peaty sediment of the 128–30 cm represents the period from 9 to 0.3 ka BP (Table 1; Fig 1). Generally, the interval from 9 to 2 ka BP displayed much lower sedimentation rates than the period from 2 to 0.3 ka BP (Fig 1).

**Table 1:** Results of the ^14^C AMS dating from the SWGT peat core, southern China.

Sample name	Test organization	AMS code	Depth (cm)	^14^C (yr BP)	Calibrated age (cal. yr BP)
GT-4-16	Beta Analytic, Inc.	440017	31	260 ± 30	150–430
GT-4-25	Arizona AMS Laboratory	AA99846	49	1130±40	960–1170
GT-4-35	Beta Analytic, Inc.	341149	69	1810±30	1630–1820
GT-4-42	Beta Analytic, Inc.	430609	83	2030±30	1900–2100
GT-4-50	Arizona AMS Laboratory	AA99847	99	4130±40	4530–4820
GT-4-55	Beta Analytic, Inc.	341150	109	6180±30	6990–7170
GT-4-60	Beta Analytic, Inc.	341151	119	7220±40	7960–8160
GT-4-64	Arizona AMS Laboratory	AA99848	127	8070±40	8780–9112

All dates shown were obtained from bulk peat using a ^14^C accelerator mass spectrometer. Calibrated age ranges were obtained using the Calib 7.0 software with 2 σ error intervals [32].

### *n*-Alkane distributions

**Fig 2:**
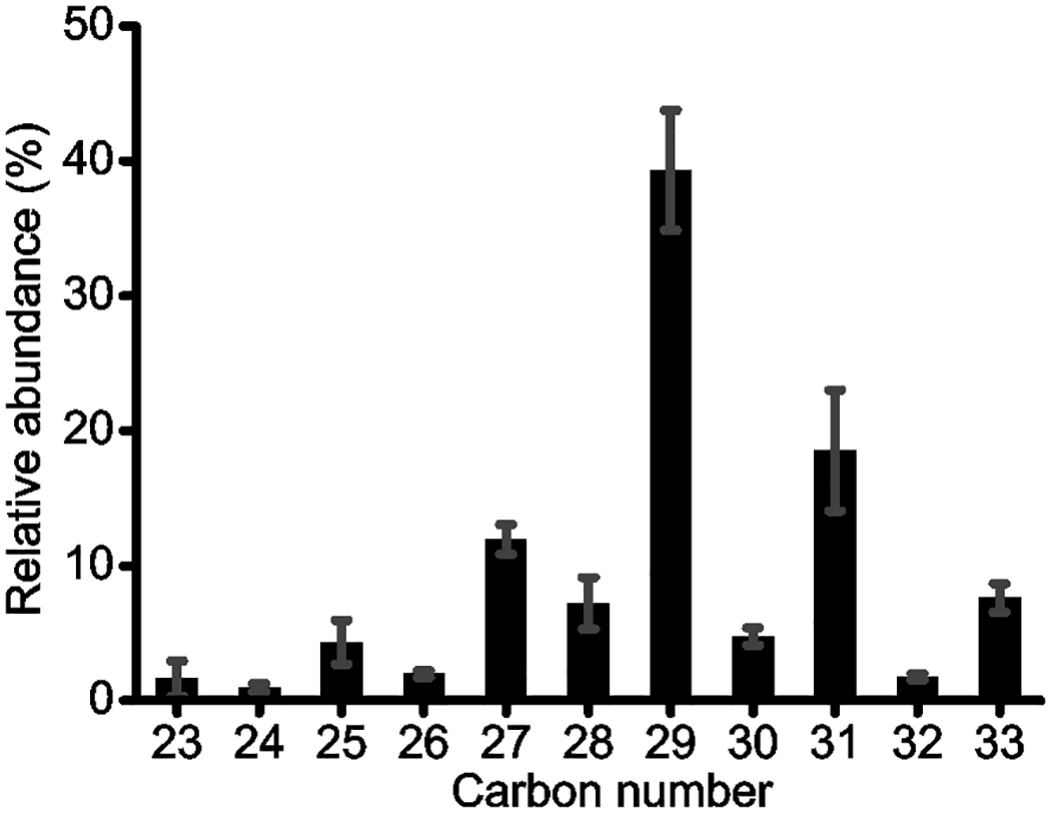
The averaged histogram of long-chain *n*-alkanes in the SWGT peat core.

In all peat samples, *n*-C_23-33_ alkanes were present with a strong odd over even predominance (Fig 2), which is indicative of a predominant contribution of higher plants to the peat [33]. The *n*-C_29_ alkane was the most abundant homologue, followed by the *n*-C_31_ alkane (Fig 2). On average, *n*-C_29_ and *n*-C_31_ alkanes accounted for around 40% and 20% of the total *n*-alkane concentrations, respectively (Fig 2).

**Fig 3:**
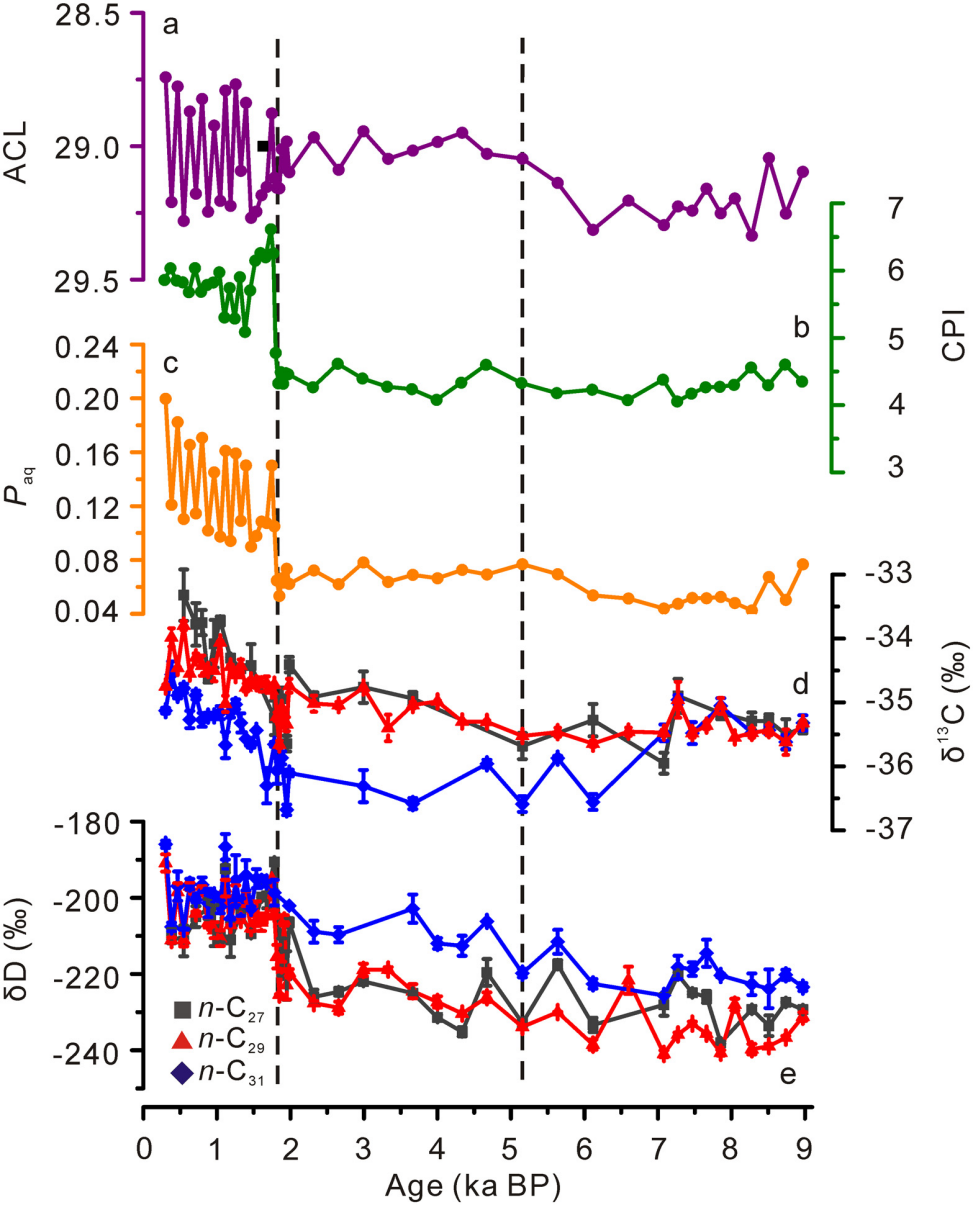
Downcore profiles of *n*-alkane proxies in the SWGT peat core. (a) ACL, (b) CPI, (c) *P*_aq_, (d) δ^13^C values of individual *n*-alkanes (square, *n*-C_27_; triangle, *n*-C_29_; round, *n*-C_31_), and (e) δD values of individual *n*-alkanes (square, *n*-C_27_; triangle, *n*-C_29_; diamond, *n*-C_31_). ACL = ∑(n×C_n_) / ∑C_n_ (23≤n≤33); CPI = (∑odd C_23_-C_31_ + ∑odd C_25_-C_33_) /2 / ∑even C_24_-C_32_; *P*_aq_ = (C_23_ + C_25_)/(C_23_ + C_25_ + C_29_ + C_31_).

The ACL values of long-chain *n*-alkanes fluctuated slightly from 29.1 to 29.3 during 9–5 ka BP (Fig 3). Values remained around 29.0 from 5 to 1.8 ka BP. After that, the ACL values varied dramatically between 28.7 and 29.3 (Fig 3). The CPI values of long-chain *n*-alkanes varied between 4.1 and 4.6 from 9 to 1.8 ka BP (Fig 3). They began to increase at 1.8 ka BP and remained around 5.5 afterwards (Fig 3). *P*_aq_ values kept relatively low between 0.04 and 0.08 during 9–1.8 ka BP (Fig 3). They started to increase at 1.8 ka BP and finally reached the peak of 0.2 at 0.3 ka BP (Fig 3).

### Compound-specific carbon and hydrogen isotope compositions of *n*-alkanes

The δ^13^C and δD values of *n*-C_27_, *n*-C_29_, *n*-C_31_ alkanes were determined. Due to the relatively low concentrations, some data are not available in the *n*-C_27_ and *n*-C_31_ alkane isotope records. *n*-C_27_, *n*-C_29_ and *n*-C_31_ alkane δ^13^C (δ^13^C_C27_, δ^13^C_C29_ and δ^13^C_C31_, respectively) values ranged between -36.0‰ and -33.3‰, -35.7‰ and -33.8‰ and -36.7‰ and -34.5‰, respectively, with δ^13^C_C31_ values generally more depleted than δ^13^C_C27_ and δ^13^C_C29_ values (Fig 3). Over the last 9 ka, δ^13^C_C29_ values showed a stronger correlation with δ^13^C_C27_ values (correlation coefficient *r* = 0.93, *p* < 0.05) than with δ^13^C_C31_ values (*r* = 0.79, *p* < 0.05).

*n*-C_27_, *n*-C_29_ and *n*-C_31_ alkane δD values (δD_C27_, δD_C29_ and δD_C31_, respectively) varied between -238‰ and -191‰, -241‰ and -191‰ and -226‰ and -186‰, respectively and showed similar covariances (*r* = 0.90, *p* < 0.05) among each other (Fig 3). Notably, δD_C31_ values were enriched in D relative to δD_C27_ and δD_C29_ values until around 1.8 ka BP (Fig 3). Our study mainly focuses on the isotope compositions of *n*-C_29_ alkane as the more concentrated compound may generate a more robust signal during δ^13^C and δD measurements.

**Fig 4:**
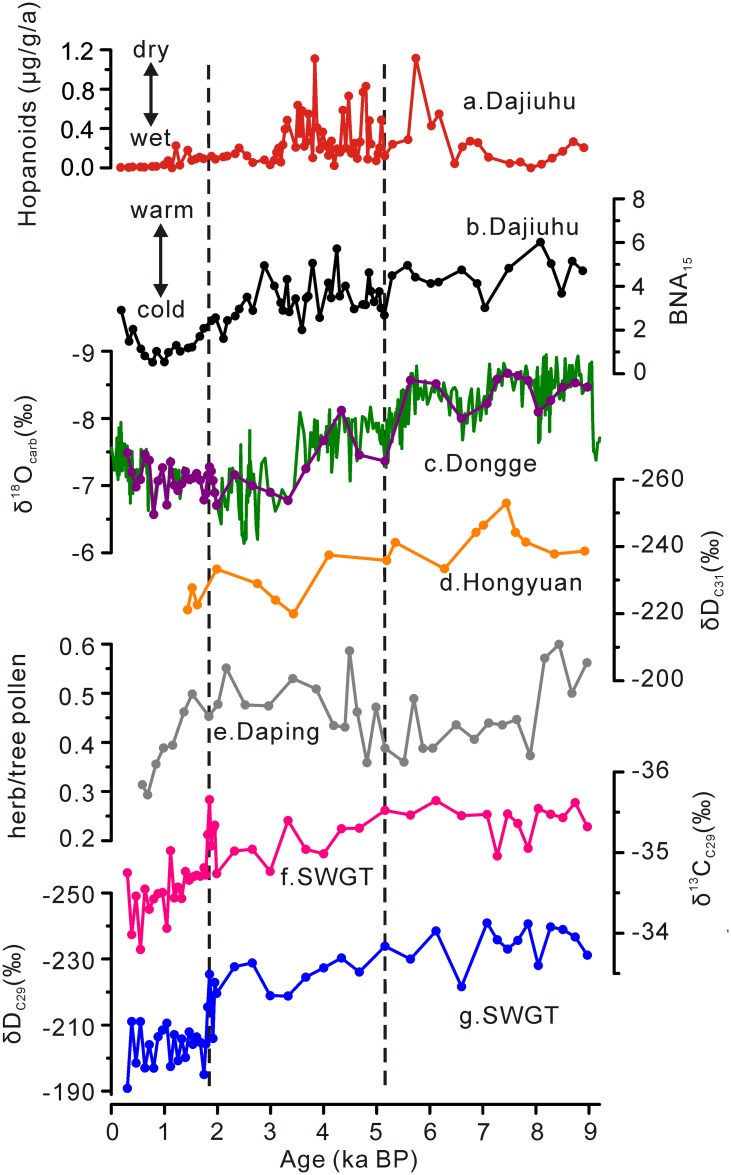
Comparisons of records. (a) Mass accumulation rate of hopanoids in the Dajiuhu peatland [26]. (b) BNA_15_ record from the Dajiuhu peatland[24], (c) δ^18^O_carb_ record from Dongge Cave [30], (d) δD_C31_ record from the Hongyuan peat sequence [19], (e) ratios of total herb pollen over total tree pollen from the Daping sedimentary sequence [29], (f) δ^13^C _C29_ record (this study) and (g) δD_C29_ record (this study).

In detail, the δ^13^C_C29_ values varied between -35.6‰ and -35.0‰ during 9–5 ka BP (Fig 4). They showed slightly more positive values between -34.7‰ and -35.7‰ during 5–1.8 ka BP (Fig 4). After 1.8 ka BP, δ^13^C_C29_ values exhibited a more positive trend with an amplitude up to 2‰ (Fig 4). δD_C29_ values displayed an overall increasing trend from 9 to 1.8 ka BP with values ranging between -241‰ and -206‰ (Fig 4). δD_C29_ values showed a remarkable increase from -225‰ to -195‰ around 1.8 ka BP (Fig 4). After that, they fluctuated around -200‰ (Fig 4).

## Discussion

### Interpretations of the *n*-alkane proxies

#### ACL

The ACL values describe the average number of carbon atoms based on the concentrations of long-chain *n*-alkane homologues [34]. Though humidity and plant types may also influence *n*-alkane ACL values [35, 36], numerous studies explain the variations of *n*-alkane ACL values from the perspective of temperature, with larger ACL values indicative of warmer climates and vice versa (e.g. [34, 37–40). Such an interpretation has been validated in the Dahu peat sequence, south China [28].

#### CPI

*n*-Alkane CPI values express the predominance of odd-numbered over even-numbered *n*-alkanes [41]. Higher plants normally have CPI values >4. *n*-Alkanes from bacteria and algae show CPI values around 1 [42, 43]. Degraded or matured organic matter is also characterized by low CPI values, due to microbial reworking and cracking, respectively. These processes enable a paleoclimatic interpretation of the CPI under certain conditions. For example, if microbial reworking of peat is suppressed under cold and dry conditions, higher CPI values will be preserved in sediments [6, 44].

#### *P*_aq_

Ficken et al. (2000) [45] proposed the *P*_aq_ index ((C_23_ + C_25_)/(C_23_ + C_25_ + C_29_ + C_31_) *n*-alkanes) to reflect the relative contributions of aquatic macrophytes and terrestrial plants to lake sediments. As *Sphagnum* species have *n*-alkane distributions similar to those of aquatic macrophytes [46–48], Nichols et al. (2006) [49] proposed this proxy to reflect the relative abundance of *Sphagnum* over other bog-forming plants in boreal peatlands. In the SWGT peat core, the *P*_aq_ values are always <0.2, which excludes the possibility that *Sphagnum* species are an important contributor to *n*-alkanes preserved in this core. During the interval of 1.8–0.3 ka, the increased trend of *P*_aq_ values suggests that the contribution from *Sphagnum* species rises a little during this interval. In present, *Sphagnum* species and vascular species dominate in the SWGT peatland.

#### δ^13^C values of long-chain *n*-alkanes

Sedimentary plant wax δ^13^C values are often used to determine relative contributions of plants with different photosynthetic pathway, i.e. C_3_ and C_4_ plants [50, 51]. The CO_2_ fixation pathways used by most terrestrial plants yield distinct *n*-alkane δ^13^C values, which range from -28‰ to -40‰ in C_3_ plants and -17‰ to -25‰ in C_4_ plants [50, 51]. Peatlands are waterlogged conditions, which do not favor the growth of C_4_ plants. In the SWGT peat core, the δ^13^C signatures of long chain *n*-alkanes revealed an overwhelming predominance of C_3_ plants. In addition, the consistent patterns of *n*-alkanes in the whole core do not support that vegetation changes played an important factor on the fluctuations of *n*-alkane δ^13^C values in SWGT. Previous studies have demonstrated that δ^13^C values from peat organic matter in the monsoon regions of China can be an indicator of regional relative humidity (RH) [5, 16, 52]. Under relatively dry conditions, peat-forming vascular plants can close their stomata to reduce leaf evapotranspiration and prevent the excessive loss of water. This will also make less CO_2_ enter into the leaves through the stomata, which will lead to weaker discrimination against ^13^CO_2_ and result in less depleted δ^13^C values [53, 54]. As such, we interpret *n*-alkane δ^13^C values from SWGT peatland as a proxy for RH, with more positive δ^13^C values under dry climate.

#### δD values of long-chain *n*-alkanes

Globally, sedimentary plant wax δD values reliably track the precipitation δD values [55]. In addition, soil water evaporation and leaf transpiration can modify the primary signal, especially in arid environments [56, 57]. Furthermore, biosynthetic fractionation between source water and plant wax varies greatly among different life forms [57] and is not necessarily constant even for the same plant species [58]. Because the magnitudes of these influencing factors in different environments are not well constrained, the interpretation of leaf wax δD values often depends on site specific conditions. On the other hand, sedimentary accumulations of plant waxes tend to average and smoothen these effects which take place on the level of individual plants, enabling the use of plant wax δD records as robust paleohydrological records [55].

**Fig 5:**
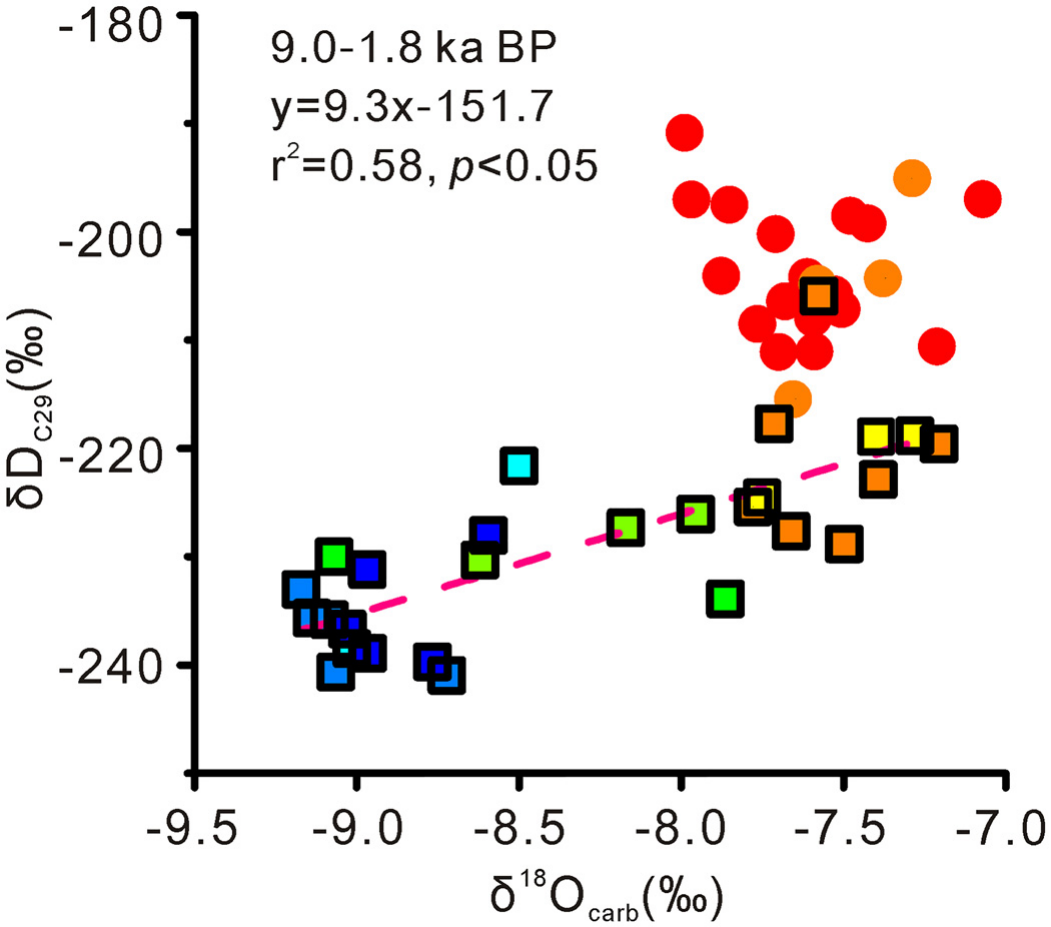
Correlation between δD_C29_ values of the SWGT peat core and δ^18^O_carb_ values of Dongge Cave [30]. Square, 9–1.8 ka BP; round, 1.8–0.3 ka BP. Symbol colors represent data age.

During 9 to 1.8 ka BP, the δD_C29_ values exhibited a general more positive trend in the SWGT peat core, which is coherent with the δD_C31_ record from the Hongyuan peat core in southwest China [19] (Fig 4). Furthermore, our δD_C29_ record and δ^18^O_carb_ record from the nearby Dongge Cave [30] showed a significant correlation during this period (*r* = 0.76, *p* < 0.05) (Fig 5) indicating a common driver of variability. Stalagmite δ^18^O records in China are thought to primarily reflect isotope composition of the moisture source [59]. Therefore, the δD_C29_ values in our record are considered to primarily record moisture source δD signals, which is in agreement with the previous studies in other localities in the monsoonal region of China [19, 60]. Due to the close proximity of these two records and their apparent similarity, we can assume that both sites received moisture from the same source. This allows us to evaluate moisture source δD and δ^18^O values in concert. During the period from 9.0 and 1.8 ka, the regression function of δD_C29_ and δ^18^O_carb_ values showed a slope of 9.3, which is slightly steeper that the slope of the modern regional meteoric water line (δD = 8.9×δ^18^O+15.5) in the nearby Guilin City (~100 km away) [61]. This may result from the potential effect of evapotranspiration on the δD values of leaf wax *n*-alkanes (which would not act upon stalagmite δ^18^O values).

However, during the period from 1.8 to 0.3 ka BP, the correlation between δD_C29_ values and δ^18^O_carb_ values was absent (Fig 5), with values falling above the regression line from the previous period (Fig 4). This may arise from an enhanced influence of evapotranspiration on leaf wax δD signatures [57] in drier conditions. Besides, an influence of vegetation changes on δD values is reflected by the similar δD_C27_, δD_C29_ and δD_C31_ values during this interval, which are not observed during 9–1.8 ka BP (Fig 4). This vegetation effect is also suggested by *P*_aq_ values, which imply an increasing input of *Sphagnum* during this interval. *Sphagnum* tends to take up the surface water while the vascular plants make use of deeper water through root system. Therefore, the *n*-alkane δD values of *sphagnum* may be more positive than those of vascular plants growing on the same site [62]. Another possibility is a change to more arid/warm moisture source.

### Paleoclimate changes in SWGT over the last 9 ka

Based on our new biomarker and stable isotope records from the SWGT peat core, the climate evolution over the last 9 ka can be divided into three stages.

During the first stage (9–5 ka BP), more negative δ^13^C_C29_ values reveal a wet condition (Fig 4). Higher ACL values agree with the higher temperatures in the Dajiuhu record, central China [24] (Figs 3 and 4). Lower CPI values imply wet and warm climate (Fig 3). Quite low *P*_aq_ values may be indicative of little contribution from *Sphagnum* (Fig 3). Together, they reveal that this period is featured by warm and wet climate. Such a period of higher temperature and precipitation broadly coincides with the Holocene optimum reported from the pollen data from the nearby Daping Swamp (8.2–4.6 ka BP; [29, 63]), and the Dahu peat deposit (26°46′N, 119°02′E; 1007 m a.s.l.) (~9–6 ka BP; [28]), and Gantang peat (31°29′N, 109°59′E; 1750 m a.s.l.) (8.2–4 ka BP; [64]). Also, our results do not support the deduction that the Holocene optimum occurred at ~3 ka in south China [1].

During the second stage (5–1.8 ka BP), the less negative δ^13^C_C29_ values imply a shift to a drier climate (Fig 4). CPI and *P*_aq_ values showed few changes (Fig 3). The decreased ACL values followed the pattern of temperature changes in Dajiuhu [24], suggesting lower temperatures (Figs 3 and 4). We consider the climate during this stage as relatively cool and dry. This is supported by the pollen record from the Daping Swamp, which suggests a shift to cool and dry climate at 4.5 ka BP [29, 63] (Fig 4). A decrease in moisture during this period has been widely reported in various areas in China, India and Africa and has been considered as a crucial cause of widespread cultural collapse in low-latitude regions [9, 65–68].

After 1.8 ka BP, both δD_C29_ and δ^13^C_C29_ values showed marked positive excursions, probably linked with dry climate (Fig 4). The ACL values on average displayed lower values, agreeing with the lower temperatures in Dajiuhu [24] (Figs 3 and 4). The CPI values increased greatly, which is indicative of cold and dry climate (Fig 3). The higher *P*_aq_ values suggest more contribution from *Sphagnum* due to the cool or/and wet condition (Fig 3). Together, they indicate an abrupt change to colder and drier climate from 1.8 ka BP onwards. The bulk dry density in the nearby Daping peat deposit recorded an abrupt decrease at 1.7 ka [63]. Calcite oxygen isotope composition from Dongge Cave stalagmite DA inferred a weak monsoon interval centered at 1.6 ka [69]. Such a sudden change to dry climate has also been reported from Lake Huguangyan [70]. However, we cannot rule out the possible influence from human activity on the environmental change around 1.8 ka BP, as agriculture developed rapidly in southern China from approximately 2 ka BP to present [67, 71]. Besides, the influence from root percolation on the upper peat layer is possible.

Comparing with the first and second stages, the interval between 1.8 and 0.3 ka has a much higher sedimentation rate (Fig 1). The alkane proxies deduced cooler and drier climate during 1.8–0.3 ka would constrain microbial degradation and facilitate organic matter accumulation. In addition, the vegetation types would also have an influence on the degradation. *Sphagnum* species generally have a slow decay rate than vascular peat-forming plants [72]. It is puzzling that alkane proxies particularly ACL and *P*_aq_ show remarkable fluctuations during 1.8–0.3 ka. Future work is required to testify the possible causes for such a large and frequent change of alkane ratios in this peat core under a drier and cooler climate during 1.8–0.3 ka.

Our record reveals a warm and wet period from 9 to 5 ka BP, followed by a transition of a relatively cool and dry condition from 5 to 1.8 ka BP, and turned to cold and dry condition from 1.8 ka BP onwards. This climate pattern corresponds to the broad decline in Asian Monsoon intensity through the latter part of the Holocene, which is linked with the orbitally induced lowering of Northern Hemisphere summer solar insolation and the associated southward migration of the mean position of Intertropical Convergence Zone [73, 74]. Our results do not support the assertion that the Holocene optimum occurred around 3 ka BP in south China [1].

## Conclusions

We analyzed the distributions and the δ^13^C and δD values of long-chain *n*-alkanes in a peat core from the SWGT peatland, south China over the last 9 ka. With these alkane ratios, climate evolution during the last 9 ka in the SWGT peatland can be divided into three stages. The first stage (9–5 ka BP) was warm and wet, which is regarded as the Holocene optimum. The second stage (5–1.8 ka BP) witnessed a shift to relatively cool and dry climate. During the third stage (1.8–0.3 ka BP), an abrupt change to drier and colder condition occurred. This climate pattern corresponds to the broad decline in Asian Monsoon intensity through the latter part of the Holocene. Our results do not support the assertion that the Holocene optimum occurred later in south China.

## Supporting Information

S1 Table Total concentrations (μg/g) and ACL, CPI and *P*_aq_ values of *n*-alkanes in the Shiwangutian peat core. (DOCX)

S2 Table δ^13^C values of *n*-alkanes in the Shiwangutian peat core. (DOCX)

S3 Table δD values of *n*-alkanes in the Shiwangutian peat core. (DOCX)
