# Correction: Molecular Paleoclimate Reconstructions over the Last 9 ka from a Peat Sequence in South China

**DOI:** 10.1371/journal.pone.0163650

**Published:** 2016-09-21

**Authors:** Xinxin Wang, Xianyu Huang, Dirk Sachse, Weihua Ding, Jiantao Xue

In Fig 4, the curve of hopanoids from a. Dajiuhu peat was plotted incorrectly. The y-axis scale of oxygen isotope values from c. Dongge should shift up by .5, so that the lower value is 6.5 and the upper value is 9.5. Please see the corrected Fig 4 here.

**Fig 4:**
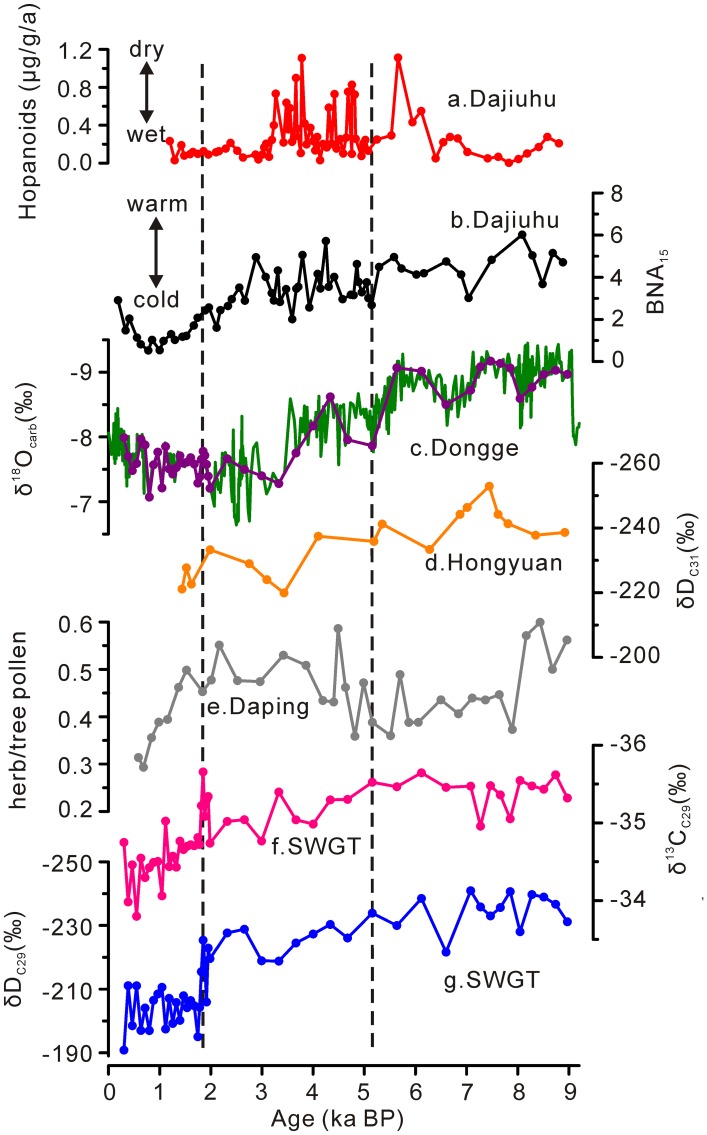
Comparisons of records. (a) Mass accumulation rate of hopanoids in the Dajiuhu peatland [26]. (b) BNA15 record from the Dajiuhu peatland[24], (c) δ18Ocarb record from Dongge Cave [30], (d) δDC31 record from the Hongyuan peat sequence [19], (e) ratios of total herb pollen over total tree pollen from the Daping sedimentary sequence [29], (f) δ13C C29 record (this study) and (g) δDC29 record (this study).
