# Supplementary material for: Molecular Paleoclimate Reconstructions over the Last 9 ka from a Peat Sequence in South China
Source: PLoS One. 2016 Aug 9;11(8):e0160934. doi: 10.1371/journal.pone.0160934 (PMC4978407; doi:10.1371/journal.pone.0160934)
Supplement: S1 Table — (DOCX) [file pone.0160934.s001.docx]

S1 Table. Total concentrations (μg/g) and ACL, CPI and Paq values of n-alkanes in the Shiwangutian peat core.

| depth | age | total | ACL | CPI | *P*aq |
| --- | --- | --- | --- | --- | --- |
| (cm) | (a BP) | (μg/g) |  |  |  |
| 31 | 301 | 125.3 | 28.74 | 5.86 | 0.20 |
| 33 | 383 | 248.1 | 29.21 | 6.04 | 0.12 |
| 35 | 466 | 136.4 | 28.78 | 5.85 | 0.18 |
| 37 | 549 | 255.5 | 29.28 | 5.83 | 0.11 |
| 39 | 632 | 115.1 | 28.87 | 5.68 | 0.17 |
| 41 | 715 | 241.7 | 29.18 | 6.04 | 0.11 |
| 43 | 798 | 145.5 | 28.82 | 5.69 | 0.17 |
| 45 | 880 | 267.1 | 29.25 | 5.79 | 0.10 |
| 47 | 963 | 166.7 | 28.92 | 5.82 | 0.15 |
| 49 | 1046 | 229.0 | 29.20 | 5.97 | 0.10 |
| 51 | 1116 | 99.0 | 28.79 | 5.30 | 0.16 |
| 53 | 1186 | 258.1 | 29.22 | 5.74 | 0.09 |
| 55 | 1256 | 105.0 | 28.77 | 5.29 | 0.16 |
| 57 | 1326 | 235.9 | 29.09 | 5.90 | 0.11 |
| 59 | 1396 | 157.0 | 28.84 | 5.09 | 0.15 |
| 61 | 1466 | 291.4 | 29.27 | 5.70 | 0.09 |
| 63 | 1537 | 291.0 | 29.25 | 6.15 | 0.10 |
| 65 | 1607 | 245.5 | 29.18 | 6.26 | 0.11 |
| 67 | 1677 | 280.2 | 29.15 | 6.20 | 0.11 |
| 69 | 1747 | 218.0 | 28.88 | 6.61 | 0.15 |
| 71 | 1781 | 368.7 | 29.12 | 6.26 | 0.11 |
| 73 | 1815 | 371.4 | 29.16 | 4.78 | 0.06 |
| 75 | 1849 | 443.5 | 29.16 | 4.32 | 0.05 |
| 77 | 1882 | 276.8 | 29.01 | 4.49 | 0.07 |
| 79 | 1916 | 382.7 | 29.08 | 4.32 | 0.06 |
| 81 | 1950 | 256.3 | 28.98 | 4.47 | 0.07 |
| 83 | 1984 | 418.6 | 29.10 | 4.46 | 0.06 |
| 85 | 2320 | 296.1 | 28.97 | 4.26 | 0.07 |
| 87 | 2656 | 420.6 | 29.09 | 4.62 | 0.06 |
| 89 | 2993 | 288.1 | 28.94 | 4.40 | 0.08 |
| 91 | 3329 | 422.0 | 29.05 | 4.27 | 0.06 |
| 93 | 3665 | 309.8 | 29.02 | 4.24 | 0.07 |
| 95 | 4001 | 186.6 | 28.98 | 4.08 | 0.07 |
| 97 | 4337 | 269.7 | 28.95 | 4.33 | 0.07 |
| 99 | 4673 | 316.8 | 29.03 | 4.60 | 0.07 |
| 101 | 5155 | 188.2 | 29.05 | 4.33 | 0.08 |
| 103 | 5636 | 161.0 | 29.14 | 4.18 | 0.07 |
| 105 | 6117 | 178.6 | 29.31 | 4.23 | 0.05 |
| 107 | 6599 | 167.4 | 29.20 | 4.07 | 0.05 |
| 109 | 7080 | 266.3 | 29.30 | 4.38 | 0.04 |
| 111 | 7273 | 204.8 | 29.23 | 4.05 | 0.05 |
| 113 | 7467 | 375.3 | 29.24 | 4.17 | 0.05 |
| 115 | 7660 | 235.3 | 29.16 | 4.26 | 0.05 |
| 117 | 7853 | 269.6 | 29.25 | 4.27 | 0.05 |
| 119 | 8046 | 266.0 | 29.20 | 4.30 | 0.05 |
| 121 | 8277 | 360.5 | 29.34 | 4.56 | 0.04 |
| 123 | 8509 | 161.3 | 29.04 | 4.29 | 0.07 |
| 125 | 8740 | 307.6 | 29.25 | 4.60 | 0.05 |
| 127 | 8971 | 95.0 | 29.10 | 4.35 | 0.08 |
