# Supplementary material for: Molecular Paleoclimate Reconstructions over the Last 9 ka from a Peat Sequence in South China
Source: PLoS One. 2016 Aug 9;11(8):e0160934. doi: 10.1371/journal.pone.0160934 (PMC4978407; doi:10.1371/journal.pone.0160934)
Supplement: S2 Table — (DOCX) [file pone.0160934.s002.docx]

S2 Table. δ^13^C values of *n*-alkanes in the Shiwangutian peat core.

| depth | age | δ^13^C_C27_ | error bar | δ^13^C_C29_ | error bar | δ^13^C_C31_ | error bar |
| --- | --- | --- | --- | --- | --- | --- | --- |
| (cm) | (a BP) | (‰) | (‰) | (‰) | (‰) | (‰) | (‰) |
| 31 | 301 | n.a.^*^ | n.a. | -34.7 | 0.0 | -35.1 | 0.0 |
| 33 | 383 | n.a. | n.a. | -34.0 | 0.1 | -34.6 | 0.2 |
| 35 | 466 | n.a. | n.a. | -34.5 | 0.0 | -34.9 | 0.0 |
| 37 | 549 | -33.3 | 0.4 | -33.8 | 0.1 | -34.8 | 0.1 |
| 39 | 632 | n.a. | n.a. | -34.5 | 0.0 | -35.3 | 0.1 |
| 41 | 715 | -33.8 | 0.3 | -34.3 | 0.1 | -34.9 | 0.1 |
| 43 | 798 | -33.8 | 0.2 | -34.4 | 0.2 | -35.3 | 0.1 |
| 45 | 880 | -34.6 | 0.1 | -34.5 | 0.0 | -35.2 | 0.1 |
| 47 | 963 | -34.1 | 0.4 | -34.5 | 0.2 | -35.2 | 0.0 |
| 49 | 1046 | -33.7 | 0.1 | -34.1 | 0.0 | -35.2 | 0.1 |
| 51 | 1116 | n.a. | n.a. | -35.0 | 0.1 | -35.7 | 0.2 |
| 53 | 1186 | -34.3 | 0.0 | -34.4 | 0.0 | -35.2 | 0.1 |
| 55 | 1256 | n.a. | n.a. | -34.6 | 0.0 | -35.0 | 0.1 |
| 57 | 1326 | -34.5 | 0.1 | -34.4 | 0.1 | -35.3 | 0.1 |
| 59 | 1396 | n.a. | n.a. | -34.8 | 0.1 | -35.6 | 0.0 |
| 61 | 1466 | -34.4 | 0.3 | -34.7 | 0.0 | -35.7 | 0.0 |
| 63 | 1537 | n.a. | n.a. | -34.7 | 0.1 | -35.4 | 0.0 |
| 65 | 1607 | n.a. | n.a. | -34.7 | 0.1 | n.a. |  |
| 67 | 1677 | n.a. | n.a. | -34.7 | 0.1 | -36.3 | 0.3 |
| 69 | 1747 | n.a. | n.a. | -34.8 | 0.0 | n.a. | n.a. |
| 71 | 1781 | -35.2 | 0.3 | -34.7 | 0.0 | -35.7 | 0.0 |
| 73 | 1815 | n.a. | n.a. | -35.2 | 0.1 | -36.1 | 0.2 |
| 75 | 1849 | n.a. | n.a. | -35.7 | 0.1 | n.a. | n.a. |
| 77 | 1882 | -34.9 | 0.2 | -35.1 | 0.0 | -35.9 | 0.1 |
| 79 | 1916 | n.a. | n.a. | -35.3 | 0.1 | n.a. | n.a. |
| 81 | 1950 | -35.6 | 0.1 | -35.3 | 0.1 | -36.7 | 0.1 |
| 83 | 1984 | -34.4 | 0.1 | -34.7 | 0.1 | -36.1 | 0.1 |
| 85 | 2320 | -34.9 | 0.1 | -35.0 | 0.1 | n.a. | n.a. |
| 87 | 2656 | n.a. | n.a. | -35.0 | 0.1 | n.a. | n.a. |
| 89 | 2993 | -34.8 | 0.2 | -34.8 | 0.0 | -36.3 | 0.3 |
| 91 | 3329 | n.a. | n.a. | -35.4 | 0.2 | n.a. | n.a. |
| 93 | 3665 | -34.9 | 0.1 | -35.0 | 0.1 | -36.6 | 0.1 |
| 95 | 4001 | n.a. | n.a. | -35.0 | 0.0 | n.a. | n.a. |
| 97 | 4337 | n.a. | n.a. | -35.3 | 0.0 | n.a. | n.a. |
| 99 | 4673 | n.a. | n.a. | -35.3 | 0.0 | -36.0 | 0.1 |
| 101 | 5155 | -35.7 | 0.2 | -35.5 | 0.0 | -36.6 | 0.1 |
| 103 | 5636 | n.a. | n.a. | -35.5 | 0.1 | -35.9 | 0.0 |
| 105 | 6117 | -35.3 | 0.3 | -35.6 | 0.0 | -36.6 | 0.1 |
| 107 | 6599 | n.a. | n.a. | -35.5 | 0.1 | n.a. | n.a. |
| 109 | 7080 | -36.0 | 0.2 | -35.5 | 0.0 | -36.2 | 0.1 |
| 111 | 7273 | -34.9 | 0.3 | -35.0 | 0.3 | -35.9 | 0.1 |
| 113 | 7467 | n.a. | n.a. | -35.5 | 0.1 | -36.1 | 0.2 |
| 115 | 7660 | n.a. | n.a. | -35.4 | 0.1 | n.a. | n.a. |
| 117 | 7853 | -35.2 | 0.1 | -35.1 | 0.1 | -35.9 | 0.1 |
| 119 | 8046 | n.a. | n.a. | -35.5 | 0.0 | n.a. | n.a. |
| 121 | 8277 | -35.3 | 0.1 | -35.5 | 0.1 | -36.2 | 0.0 |
| 123 | 8509 | -35.3 | 0.1 | -35.4 | 0.1 | -35.9 | 0.1 |
| 125 | 8740 | -35.5 | 0.2 | -35.6 | 0.2 | -35.8 | 0.1 |
| 127 | 8971 | -35.4 | 0.1 | -35.3 | 0.0 | -35.8 | 0.1 |

^*^: not available.
