# Supplementary material for: Molecular Paleoclimate Reconstructions over the Last 9 ka from a Peat Sequence in South China
Source: PLoS One. 2016 Aug 9;11(8):e0160934. doi: 10.1371/journal.pone.0160934 (PMC4978407; doi:10.1371/journal.pone.0160934)
Supplement: S3 Table — (DOCX) [file pone.0160934.s003.docx]

S3 Table. δD values of *n*-alkanes in the Shiwangutian peat core.

| depth | age | δD_C27_ | error bar | δD_C29_ | error bar | δD_C31_ | error bar |
| --- | --- | --- | --- | --- | --- | --- | --- |
| (cm) | (a BP) | (‰) | (‰) | (‰) | (‰) | (‰) | (‰) |
| 31 | 301 | n.a.^*^ | n.a. | -191 | 2.3 | -186 | 1.0 |
| 33 | 383 | -210 | 1.9 | -211 | 0.7 | -208 | 1.1 |
| 35 | 466 | n.a. | n.a. | -198 | 2.3 | -197 | 3.8 |
| 37 | 549 | -211 | 4.0 | -211 | 1.7 | -208 | 0.2 |
| 39 | 632 | n.a. | n.a. | -197 | 1.0 | -196 | 1.4 |
| 41 | 715 | -205 | 2.7 | -204 | 0.5 | -201 | 0.7 |
| 43 | 798 | n.a. | n.a. | -197 | 0.0 | -197 | 1.9 |
| 45 | 880 | -203 | 2.6 | -206 | 1.3 | -201 | 3.8 |
| 47 | 963 | -207 | 5.4 | -209 | 2.1 | -199 | 0.6 |
| 49 | 1046 | -210 | 1.5 | -211 | 2.0 | -202 | 1.7 |
| 51 | 1116 | -192 | 1.0 | -197 | 2.2 | -187 | 3.3 |
| 53 | 1186 | -211 | 4.5 | -207 | 0.0 | -206 | 0.1 |
| 55 | 1256 | n.a. | n.a. | -199 | 2.5 | -195 | 4.6 |
| 57 | 1326 | -204 | 0.6 | -206 | 0.6 | -202 | 2.3 |
| 59 | 1396 | n.a. | n.a. | -200 | 2.0 | -194 | 3.9 |
| 61 | 1466 | -209 | 0.1 | -208 | 0.8 | -203 | 0.4 |
| 63 | 1537 | n.a. | n.a. | -204 | 3.2 | -195 | 2.5 |
| 65 | 1607 | -200 | 0.0 | -206 | 2.3 | -196 | 1.4 |
| 67 | 1677 | -199 | 3.3 | -205 | 0.1 | -196 | 0.2 |
| 69 | 1747 | n.a. | n.a. | -195 | 0.0 | n.a. | n.a. |
| 71 | 1781 | -191 | 0.5 | -204 | 1.0 | -199 | 3.4 |
| 73 | 1815 | n.a. | n.a. | -215 | 3.1 | n.a. | n.a. |
| 75 | 1849 | n.a. | n.a. | -225 | 0.1 | n.a. | n.a. |
| 77 | 1882 | -216 | 4.9 | -218 | 1.0 | n.a. | n.a. |
| 79 | 1916 | n.a. | n.a. | -206 | 1.4 | n.a. | n.a. |
| 81 | 1950 | -220 | 4.3 | -223 | 3.7 | n.a. | n.a. |
| 83 | 1984 | -206 | 1.1 | -220 | 0.9 | -202 | 0.0 |
| 85 | 2320 | -226 | 1.9 | -228 | 0.5 | -209 | 2.9 |
| 87 | 2656 | -225 | 1.0 | -229 | 1.6 | -210 | 2.0 |
| 89 | 2993 | -222 | 0.6 | -219 | 1.6 | n.a. | n.a. |
| 91 | 3329 | n.a. | n.a. | -219 | 0.7 | n.a. | n.a. |
| 93 | 3665 | -225 | 0.4 | -225 | 1.9 | -203 | 3.7 |
| 95 | 4001 | -231 | 0.7 | -227 | 1.5 | -212 | 1.4 |
| 97 | 4337 | -235 | 1.2 | -230 | 0.5 | -213 | 2.6 |
| 99 | 4673 | -220 | 3.5 | -226 | 1.3 | -206 | 0.2 |
| 101 | 5155 | -233 | 1.0 | -234 | 0.5 | -220 | 1.1 |
| 103 | 5636 | -218 | 1.2 | -230 | 0.1 | -212 | 3.2 |
| 105 | 6117 | -233 | 1.9 | -238 | 1.3 | -223 | 1.2 |
| 107 | 6599 | n.a. | n.a. | -222 | 3.6 | n.a. | n.a. |
| 109 | 7080 | -228 | 2.9 | -241 | 0.9 | -226 | 0.2 |
| 111 | 7273 | -220 | 0.6 | -236 | 0.5 | -218 | 3.0 |
| 113 | 7467 | -225 | 0.3 | -233 | 0.5 | -219 | 1.8 |
| 115 | 7660 | -226 | 1.6 | -236 | 0.1 | -215 | 3.6 |
| 117 | 7853 | -238 | 0.3 | -241 | 0.7 | -220 | 0.5 |
| 119 | 8046 | n.a. | n.a. | -228 | 1.6 | n.a. | n.a. |
| 121 | 8277 | -229 | 0.9 | -240 | 1.4 | -223 | 2.8 |
| 123 | 8509 | -234 | 2.7 | -239 | 0.6 | -224 | 5.0 |
| 125 | 8740 | -227 | 0.2 | -237 | 0.4 | -220 | 1.3 |
| 127 | 8971 | -229 | 0.0 | -231 | 1.1 | -223 | 1.0 |

^*^: not available.
